# Towards understanding the de-adoption of low-value clinical practices: a scoping review

**DOI:** 10.1186/s12916-015-0488-z

**Published:** 2015-10-06

**Authors:** Daniel J. Niven, Kelly J. Mrklas, Jessalyn K. Holodinsky, Sharon E. Straus, Brenda R. Hemmelgarn, Lianne P. Jeffs, Henry Thomas Stelfox

**Affiliations:** Department of Critical Care Medicine, University of Calgary, Calgary, Alberta T1Y 6J4 Canada; Department of Community Health Sciences, University of Calgary, Calgary, Alberta T2N 4Z6 Canada; Li Ka Shing Knowledge Institute of St. Michael’s Hospital, Toronto, Ontario M5B 1T8 Canada; Department of Medicine, University of Calgary, Calgary, Alberta T2N 4Z6 Canada

**Keywords:** Abandon, Contradict, De-adoption, De-implementation, Disinvestment, Low-value, Medical reversal, Obsolete, Reassess, Withdrawal

## Abstract

**Background:**

Low-value clinical practices are common in healthcare, yet the optimal approach to de-adopting these practices is unknown. The objective of this study was to systematically review the literature on de-adoption, document current terminology and frameworks, map the literature to a proposed framework, identify gaps in our understanding of de-adoption, and identify opportunities for additional research.

**Methods:**

MEDLINE, EMBASE, the Cochrane Central Register of Controlled Trials, the Cochrane Database of Systematic Reviews, the Cochrane Database of Abstracts and Reviews of Effects, and CINAHL Plus were searched from 1 January 1990 to 5 March 2014. Additional citations were identified from bibliographies of included citations, relevant websites, the PubMed ‘related articles’ function, and contacting experts in implementation science. English-language citations that referred to de-adoption of clinical practices in adults with medical, surgical, or psychiatric illnesses were included. Citation selection and data extraction were performed independently and in duplicate.

**Results:**

From 26,608 citations, 109 were included in the final review. Most citations (65 %) were original research with the majority (59 %) published since 2010. There were 43 unique terms referring to the process of de-adoption—the most frequently cited was “disinvest” (39 % of citations). The focus of most citations was evaluating the outcomes of de-adoption (50 %), followed by identifying low-value practices (47 %), and/or facilitating de-adoption (40 %). The prevalence of low-value practices ranged from 16 % to 46 %, with two studies each identifying more than 100 low-value practices. Most articles cited randomized clinical trials (41 %) that demonstrate harm (73 %) and/or lack of efficacy (63 %) as the reason to de-adopt an existing clinical practice. Eleven citations described 13 frameworks to guide the de-adoption process, from which we developed a model for facilitating de-adoption. Active change interventions were associated with the greatest likelihood of de-adoption.

**Conclusions:**

This review identified a large body of literature that describes current approaches and challenges to de-adoption of low-value clinical practices. Additional research is needed to determine an ideal strategy for identifying low-value practices, and facilitating and sustaining de-adoption. In the meantime, this study proposes a model that providers and decision-makers can use to guide efforts to de-adopt ineffective and harmful practices.

**Electronic supplementary material:**

The online version of this article (doi:10.1186/s12916-015-0488-z) contains supplementary material, which is available to authorized users.

## Background

Clinical practice evolves in response to scientific evidence through a process of *discovery* (novel practice introduced into clinical practice, e.g., systemic thrombolysis for acute ST-elevation myocardial infarction (STEMI) [1]), *replacement* (newer, more effective practice supplants current practice, e.g. tenecteplase superior to alteplase among patients with STEMI [2]), or *reversal* (current practice shown to be ineffective or harmful, e.g., suppression of ventricular ectopy after a myocardial infarction using encainide, flecainide, or moricizine [3]) [4]. Discovery and replacement introduce novel, beneficial therapies into clinical practice, while reversal implies that patients receive no benefit and may be at risk of harm [5]. The adoption of clinical practices that are later de-adopted imposes substantial inefficiencies on the healthcare system wherein resources that could have been dedicated to other purposes are instead devoted to a practice that was ineffective or harmful (e.g., self-monitoring of blood glucose in patients with type 2 diabetes mellitus managed without insulin) [6].

Practice reversal is common [5, 7, 8]. A recent review of articles published in a major general medical journal between 2001 and 2010 found that 27 % of original articles re-examined the efficacy of an established practice, among which 40 % found evidence for practice reversal [7]. In another review, commissioned by the Australian government’s Comprehensive Management Framework for managing their Medical Benefits Schedule, Elshaug and colleagues triangulated data from searches of the peer-reviewed literature, targeted health technology databases, and opportunistic sampling of stakeholder groups to identify 156 potentially unsafe and/or ineffective practices [8].

Medical reversal may be an unavoidable consequence of evidence-based medicine and/or early technology adoption; however, it is important that its incidence remain low given the threat that it poses to providing high-quality healthcare. It is equally important that any intervention with evidence for medical reversal be rapidly de-adopted. We were unable to identify any knowledge synthesis that systematically examined the de-adoption of established clinical practices. We conducted this scoping review to describe the literature on de-adoption, document current terminology and frameworks, map the literature to a proposed conceptual framework (Table 1), identify gaps in the understanding of this important concept, and identify opportunities for more detailed evidence syntheses and/or empirical research.

**Table 1:** Proposed framework for conceptualizing de-adoption

Phase of de-adoption	Operational definition
Identify low-value clinical practices	Ascertain which clinical practices are of low value
Facilitate the de-adoption process	Reduce the use of low-value clinical practices
Evaluate de-adoption outcomes	Evaluate the outcomes of a strategy of de-adoption
Sustain de-adoption	Prevent resurgence in use of low-value practices after their initial de-adoption

## Methods

We developed a conceptual framework for this work that employed the key features of Everett Rogers’ Innovation-Decision model to conceptualize de-adoption (Table 1) [9]. De-adoption was defined as the discontinuation of a clinical practice after it was previously adopted [9]. We followed established scoping review methodology [10, 11], and used the Preferred Reporting Items for Systematic Reviews and Meta-Analyses (PRISMA) guidelines to report the methods and results [12].

### Eligibility criteria

We included English-language citations that referred to the de-adoption of any clinical practice in adults (mean age ≥ 18 years) with medical, surgical, or psychiatric illnesses. All original and non-original quantitative and qualitative research citations were eligible; however, we excluded citations that exclusively described the *adoption* of practices or appropriateness of resource use (e.g., selected use of antimicrobials, appropriate use of surgical procedures, appropriate use of lumbar spine radiography among patients with lower back pain). Although de-adoption is a component within the larger issue of resource optimization, the “appropriateness” of a clinical practice embodies more than simply discontinuing its use. Therefore, we excluded citations primarily focused on clinical practice appropriateness.

### Search strategy and data sources

With the help of a medical librarian, we searched the following electronic databases from 1 January 1990 to 5 March 2014: Ovid MEDLINE, Ovid EMBASE, the Cochrane Central Register of Controlled Trials (CENTRAL), the Cochrane Database of Systematic Reviews, the Cochrane Database of Abstracts and Reviews of Effects, and CINAHL Plus. Pilot searches in MEDLINE suggested that none of the currently available Medical Subject Heading (MeSH) terms were specific to articles reporting de-adoption. Therefore, the MEDLINE search was confined to use of text words that included combinations and synonyms of *de-adoption* and *healthcare technologies* (Additional file 1: Appendix)*.* Search terms were combined using the appropriate Boolean logic, and included wildcards to account for plural words and variations in spelling. The search strategy included similar combinations of terms within the other databases. To ensure reproducibility, the MEDLINE search strategy was peer reviewed by a second medical librarian using the Peer Review of Electronic Search Strategies (PRESS) checklist [13].

To increase the sensitivity of the search strategy, we also searched the gray literature according to recommendations from the Canadian Agency for Drugs and Technologies in Health [14]. Relevant websites included The Canadian Agency for Drugs and Technologies in Health, Program for Assessment of Technology in Health, Australian Government Medical Services Advisory Committee, Austrian Institute of Technology Assessment, National Institute for Health and Care Excellence, Agency for Healthcare Research and Quality, Blue Cross & Blue Shield Association, Choosingwisely.org and Choosingwiselycanada.org, and the Trip Database. Additional citations were identified by (1) contacting experts in implementation science; (2) using the PubMed “related articles” function; and (3) hand-searching bibliographies from important implementation science/adoption of innovations textbooks [9, 15, 16], and reference lists of included citations. Reference management was performed in EndNote (version X7, Thomson Reuters).

### Citation selection

Prior to the screening of titles and abstracts, the citation screening form was calibrated by three team members (DJN, KJM, JKH) independently with a random sample of 50 citations. Once consistent citation selection was achieved (kappa ≥ 0.8) [17], all citations were screened for inclusion independently and in duplicate by three reviewers through a two-stage process. During level-one screening, titles and abstracts were reviewed to determine citations that met the inclusion/exclusion criteria. The full text of any citation classified as “include” or “unclear” was reviewed to determine whether it met study inclusion criteria (level-two screening). Eligibility disagreements were resolved by consensus, or arbitration by a third reviewer. Agreement between reviewers at all stages of citation selection was quantified using the kappa statistic [17].

### Data extraction and synthesis

Three reviewers independently extracted data from all included citations using a pre-designed electronic form that was pilot tested using a random sample of 10 citations. Once data were consistently abstracted (kappa ≥ 0.8) [17], reviewers proceeded with full data extraction. Extracted data pertained to (1) the citation (e.g., original research, non-original research, website); (2) the term(s) used to refer to de-adoption (e.g., discontinuance, medical reversal, rejection); (3) characteristics of the target condition(s) or clinical practice(s) (e.g., use of nesiritide in acute decompensated heart failure [18]); (4) characteristics of evidence suggesting de-adoption (e.g., original research versus non-original research); (5) whether barriers and facilitators to de-adoption were reported; and (6) whether conceptual frameworks to promote low-value practice de-adoption were used/cited.

Independently, and in duplicate, reviewers mapped the abstracted data onto the proposed conceptual framework. Articles were summarized using counts, proportions, mean (standard deviation), or median (inter-quartile range, IQR) where appropriate. Data were managed and analyzed using Stata version 13.1 (Stata Corp, College Station, TX, USA).

## Results

**Fig. 1:**
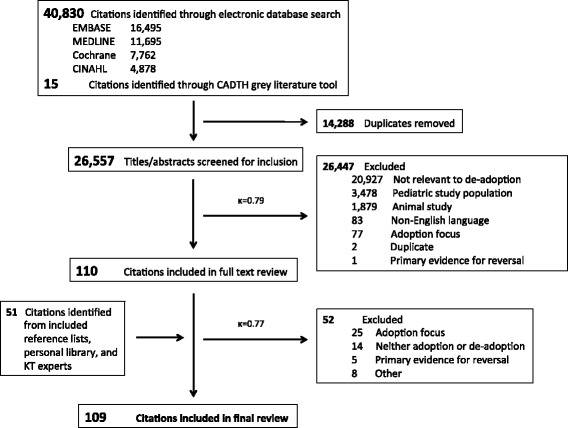
Details of the article selection process. *CADTH* Canadian Agency for Drugs and Technologies in Health, *KT* Knowledge Translation

The electronic database and gray literature searches identified 26,557 unique citations (Fig. 1) that were screened for inclusion, from which 110 full text citations were retrieved for further assessment. An additional 51 articles were identified through review of bibliographies, and consultation with knowledge translation experts. From these 161 full text citations, 109 were included in the final review. The most common reason citations were excluded after full text review was owing to an explicit focus on the adoption and/or appropriateness of clinical practices (n = 25).

### Description of the included citations

A description of the included citations is provided in Table 2. Most citations were original research studies (65 %), with the majority being either quasi-experimental (28 %) or cohort studies (14 %). Among the non-original citations, most were editorials or letters to the editor (19 %), or narrative reviews (15 %). Most articles originated in North America (60 %) with the USA representing the most common country (47 % of all articles). The majority of articles were published from 2010 onwards (59 %), with very few published prior to 2000 (3 %). Most articles described the de-adoption of therapeutic interventions (62 %), with comparatively fewer describing the de-adoption of diagnostic interventions (30 %). The randomized clinical trial was most frequently cited (41 %) as the level of evidence that should trigger de-adoption, and most articles cited risk of harm (73 %), and/or lack of efficacy (63 %) as the reason practices should be de-adopted. Among the articles that reported the original reason for clinical practice adoption (n = 16, 15 %), most (n = 10, 63 %) cited observational research (case series and cohort studies) as the evidence that shaped adoption. A detailed, referenced bibliography of the citations is provided in Additional file 1: Table S1.

**Table 2:** Characteristics of included citations

Characteristic	Number (%) of 109 citations
Year of publication	
1990–1999	3 (3)
2000–2009	42 (38)
2010–current	64 (59)
Country of origin^a^	
North America	65 (60)
Europe	30 (28)
Australasia	22 (20)
Type of article	
Original research	71 (65)
Quasi-experimental^c^	30 (28)
Cohort study^b^	15 (14)
Mixed methods	8 (7)
Qualitative	4 (4)
Predictive modeling	3 (3)
Knowledge synthesis	3 (3)
Consensus method	3 (3)
Randomized clinical trial	1 (1)
Other^d^	6 (6)
Non-original research	38 (35)
Editorial, letter to the editor, news item, other	21 (19)
Narrative review	16 (15)
Guideline	1 (1)
Focus of article	
Identify low-value practices	51 (47)
Facilitate the de-adoption process	44 (40)
Evaluate de-adoption outcomes	54 (50)
Sustain de-adoption	2 (2)
Type of intervention^e^	
Therapeutic	68 (62)
Drug	34 (31)
Device or surgical procedure	16 (15)
Drugs and devices/procedures	16 (15)
Other	3 (3)
Diagnostic	27 (30)
Laboratory	7 (8)
Physiologic measurement	4 (4)
Diagnostic imaging	3 (3)
Screening program	1 (1)
Diagnostic tests not otherwise specified	12 (13)
Evidence to promote de-adoption^f^	
Randomized clinical trial	45 (41)
Knowledge synthesis	14 (13)
Clinical practice guideline	6 (5)
Cohort study	4 (4)
Quasi-experimental^c^	2 (2)
Expert consensus	2 (2)
Reasons for de-adoption^g^	
Harm	80 (73)
Lack of efficacy	69 (63)
Not cost-effective	37 (34)

Percentages within each characteristic may not always total to 100 due to rounding error, and/or redundancy within citations (e.g., a citation may have more than one country of origin)

^a^North American countries: Canada, USA; European countries: UK, Belgium, Denmark, France, Greece, Italy, Netherlands, Spain; Australian countries: Australia, New Zealand

^b^Includes six studies wherein the study population was a cohort of articles identified through searches of the electronic literature

^c^Includes interrupted time series, and before-and-after studies

^d^Includes two surveys, one report on stakeholder engagement, one simulation

^e^Type of intervention not reported in 32 studies

^f^Not reported in 46 studies (52 %)

^g^Not reported in 11 studies

### De-adoption terminology

We identified 43 unique terms representative of the process of de-adoption (Table 3). The majority of citations (65 %) referred to de-adoption using more than one term, and among these the median (IQR) number of terms per citation was 3 (2–3). Disinvest* was the most frequently cited term (39 % of included citations). Other commonly cited terms included *decrease use* (24 %), discontinu* (16 %), abandon* (16 %), reassess* (14 %), obsole* (12 %), *medical reversal* (11 %), and contradict* (10 %). Terms such as de-implement* and de-adopt* were infrequently cited (4 % and 3 %, respectively). A term representative of the process of de-adoption was found in the title or abstract of 86 % of citations and most frequently included disinvest* (31 %), *decrease use* (12 %), reassess* (7 %), withdraw* (7 %), *medical reversal* (6 %), discontinu* (6 %), and obsole* (6 %). Each of the 43 unique terms was mapped onto our conceptual framework. The majority of terms (n = 22, 51 %) referred to facilitating the de-adoption process. Seventeen terms (40 %) mapped to more than one category within the conceptual framework, with the most common cross-classification being facilitate de-adoption and sustain de-adoption (13/17, 76 %).

**Table 3:** De-adoption terms (n = 43) and frequency of their use within included citations

Term^a^	Number (%) of 109 citations^b^	Number (%) of citations with term listed in title or abstract	Relationship to the proposed conceptual framework	References
Disinvest*	42 (39)	34 (31)	Facilitate de-adoption	[7, 8, 19, 20, 23, 42, 46–81]
			Sustain de-adoption	
Decrease use	26 (24)	13 (12)	Facilitate de-adoption	[8, 26–28, 31, 33, 35, 37, 80, 82–99]
			Evaluate de-adoption outcomes	
Discontinu*	17 (16)	7 (6)	Facilitate de-adoption	[8, 18, 20, 23, 25, 28, 51, 56, 61, 63, 91, 94, 96, 100–103]
			Evaluate de-adoption outcomes	
Abandon*	17 (16)	4 (4)	Sustain de-adoption	[31, 54, 58, 63, 65, 84, 86, 97, 98, 100, 101, 103–108]
Reassess*	15 (14)	8 (7)	Identify low-value practices	[8, 18, 23, 46, 52, 58, 59, 68, 71, 80, 102, 108–111]
Obsole*	13 (12)	6 (6)	Identify low-value practices	[19, 20, 49, 55, 57, 58, 68, 73, 76, 80, 108, 109, 112]
Medical reversal	12 (11)	7 (6)	Identify low-value practices	[4, 5, 7, 61, 83, 85, 107, 113–117]
Contradict	11 (10)	3 (3)	Identify low-value practices	[5, 7, 24, 54, 65, 68, 85, 86, 104, 118, 119]
Re-invest	9 (8)	0 (0)	Sustain de-adoption	[8, 52, 54, 55, 68, 71, 73, 78, 80]
Withdraw*	8 (7)	8 (7)	Facilitate de-adoption	[29–36]
			Sustain de-adoption	
Reduc*	8 (7)	1 (1)	Evaluate de-adoption outcomes	[31, 32, 34–36, 120, 121]
Decline in use	7 (6)	0 (0)	Evaluate de-adoption outcomes	[96, 98, 99, 103, 120, 122, 123]
Health technology reassessment	5 (5)	4 (4)	Identify low-value practices	[52, 58, 59, 71, 110]
Change in use	4 (4)	2 (2)	Evaluate de-adoption outcome	[32, 118, 121, 124]
De-implement*	4 (4)	2 (2)	Facilitate de-adoption	[50, 65, 125, 126]
De-list	4 (4)	0 (0)	Facilitate de-adoption	[57, 68, 80, 109]
			Sustain de-adoption	
Low value practice/intervention	4 (4)	2 (2)	Identify low-value practices	[7, 70, 78, 127]
Change in practice	3 (3)	1 (1)	Evaluate de-adoption outcome	[36, 103, 118]
De-adopt*	3 (3)	2 (2)	Facilitate de-adoption	[18, 100, 128]
			Evaluate de-adoption outcomes	
De-commission	3 (3)	1 (1)	Facilitate de-adoption	[68, 72, 80]
			Sustain de-adoption	
Do not do	3 (3)	1 (1)	Facilitate de-adoption	[21, 22, 56]
Reallocation	3 (3)	0 (0)	Sustain de-adoption	[58, 73, 109]
Remov*	3 (3)	0 (0)	Facilitate de-adoption	[29, 33, 37]
			Sustain de-adoption	
Replace	3 (3)	0 (0)	Facilitate de-adoption	[4, 111, 114]
			Sustain de-adoption	
Refute	3 (3)	1 (1)	Identify low-value practices	[24, 83, 104]
Over use	3 (3)	0 (0)	Identify low-value practices	[37, 127, 129]
Stop*	3 (3)	1 (1)	Facilitate de-adoption	[35, 77, 124]
Inappropriate use	2 (2)	1 (1)	Identify low-value practices	[112, 129]
Relinquish*	2 (2)	1 (1)	Facilitate de-adoption	[97, 98]
			Sustain de-adoption	
Ineffective	2 (2)	1 (1)	Identify low-value practices	[19, 29]
Misuse	1 (1)	0 (0)	Identify low-value practices	[127]
Re-appraisal	1 (1)	0 (0)	Identify low-value practices	[79]
Re-prioritization	1 (1)	0 (0)	Sustain de-adoption	[79]
Substitutional re-investment	1 (1)	0 (0)	Facilitate de-adoption	[79]
			Sustain de-adoption	
Evidence-based reassessment	1 (1)	0 (0)	Identify low-value practices	[79]
Clinical redesign	1 (1)	0 (0)	Facilitate de-adoption	[79]
Disadoption	1 (1)	0 (0)	Facilitate de-adoption	[101]
Defunding	1 (1)	0 (0)	Facilitate de-adoption	[57]
			Sustain de-adoption	
Resource release	1 (1)	0 (0)	Facilitate de-adoption	[57]
			Sustain de-adoption	
Withdrawing from a service and redeploying resources	1 (1)	0 (0)	Facilitate de-adoption	[57]
			Sustain de-adoption	
Redeploy	1 (1)	1 (1)	Facilitate de-adoption	[68]
			Sustain de-adoption	
Reversal	1 (1)	0 (0)	Identify low-value practices	[96]
			Facilitate de-adoption	
			Sustain de-adoption	
Drop in use	1 (1)	0 (0)	Facilitate de-adoption	[31]
			Evaluate de-adoption	

^a^*wildcard notation denotes multiple endings for a given term

^b^Percentages do not total 100 owing to the appearance of multiple terms within individual citations

### Barriers and facilitators to de-adoption

**Fig. 2:**
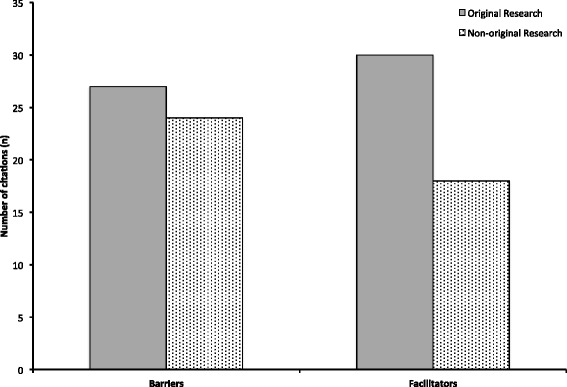
Distribution of articles citing barriers to and facilitators of de-adoption according to type of research

Barriers and facilitators to de-adoption were cited within 51 and 48 of the included citations respectively. The bulk of articles citing barriers to or facilitators of de-adoption were original research (Fig. 2).

### Mapping citations to the de-adoption conceptual framework

**Fig. 3:**
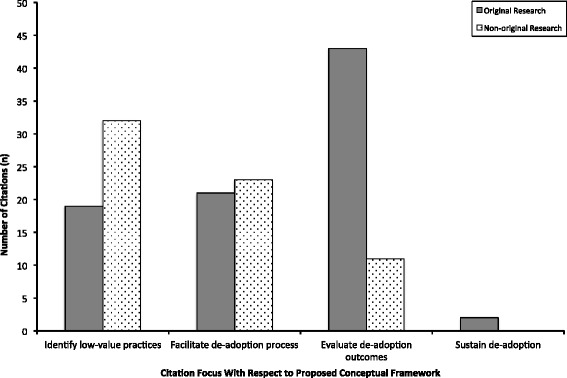
Distribution of articles according to classification within the conceptual framework and type of research

Articles frequently mapped to more than one category within our conceptual framework (Fig. 3). The primary focus among included citations was evaluating de-adoption outcomes (50 %), identifying low-value practices (47 %), and facilitating the de-adoption process (40 %). Two articles (2 %) discussed sustaining de-adoption. Most articles whose focus was on evaluating de-adoption outcomes were original research (80 %), whereas the majority of articles that discussed identifying low-value practices were non-original research (63 %).

Frameworks for the de-adoption of low-value clinical practices were provided in 11 citations (Table 4), of which half were derived from original research (n = 5, 45 %). Two citations documented clinical application of their framework [19, 20]. Seven citations described frameworks for identifying and prioritizing candidate low-value practices, and nine citations described frameworks for facilitating the de-adoption process. Among citations that described a framework for identifying low-value practices, common mechanisms included consultation with clinical stakeholders, monitoring for new scientific evidence, examining for practices with large between-provider variation, and/or embedding the notion of health technology reassessment within the life cycle of any given practice. Commonly proposed criteria for prioritizing the de-adoption of low-value practices included the availability of evidence that a candidate practice is ineffective or harmful, the safety of the low-value practice (i.e., harmful practices prioritized ahead of those that are simply ineffective), potential health and cost impact of de-adoption, and availability of alternative practices. Among citations that described frameworks for facilitating the de-adoption process, common mechanisms included restructuring of funding associated with the given practice, changes to local and/or regional policies, and more consistent integration of health technology reassessment within existing health technology assessment programs.

**Table 4:** Frameworks proposed to guide the de-adoption of low-value practices

Citation	Type of citation	Relationship to conceptual framework (Table 1)	Description	Documented clinical application
Elshaug et al. 2009 [109]	Discussion paper prepared by Canadian Agency for Drugs and Technologies in Health Health Technology Strategy Policy Forum	Identify low-value practices	Criteria for identifying existing, potentially non-cost-effective practices as candidates for assessment	No
			Criteria to inform the prioritization of candidates for detailed review after identification	
		Facilitate the de-adoption process	Funding approaches to facilitating reduction in non-cost-effective practices	No
Joshi et al. 2009 [80]	Narrative review	Identify low-value practices	HTR approach to identifying candidate technologies	No
Ibargoyen-Roteta et al. 2010 [55]	Guideline	Identify of low-value practices	GuNFT: Hospital and patient-level criteria for not funding technologies	No
		Facilitate the de-adoption process	Barriers and mechanisms to remove funding from existing technologies	No
Mortimer 2010 [68]	Narrative review	Facilitate the de-adoption process	Proposed re-orientation of traditional PBMA model to target strategies of disinvestment	Not with the re-oriented PBMA model as outlined by the authors
Donaldson et al. 2010 [72]	Narrative review	Facilitate the de-adoption process	Describes the use of PBMA to promote rational disinvestment	Not according to the model outlined by the authors
Gerdvilaite and Nachtnebel 2011 [57]	Systematic review	Identify of low-value practices	Authors cite criteria proposed by Elshaug et al. [109], Joshi et al. [80], Ibargoyen-Roteta et al. [55], and criteria proposed by National Institute for Health and Care Excellence	No
			Overlapping criteria include new evidence, cost effectiveness, safety, and available alternatives	
		Facilitate the de-adoption process	As described above for Ibargoyen-Roteta et al. [55]	No
Levin 2011 [20]	Conference presentation	Identify of low-value practices	Ontario’s Evidence-based Analyses to Manage Technology Adoption and Obsolescence: Mega-analysis Evidence Based Analyses of technologies around disease conditions; prioritized by effectiveness and cost-effectiveness; criteria for identifying practices unclear	Yes
		Facilitate the de-adoption process	Mechanism for facilitating de-adoption appears to be based on funding effective technologies, and not funding ineffective technologies	Yes
Leggett et al. 2012 [58]	Systematic review	Identify of low-value practices; Facilitate the de-adoption process	1.GuNFT as outlined above	No
			2.5-steps for HTR should include: identification, prioritization, evaluation, implementation, and monitoring	
Watt et al. 2012 [46]	Mixed methods	Facilitate the de-adoption process	Two technologies (assisted reproductive technology and vitamin B12/folate pathology tests) used as case studies to test a three-level model to facilitate de-adoption including:	No (study ongoing)
			1. Evidence reports	
			2. Stakeholder engagement	
			3. Policy deliberation and analysis; Process evaluation	
Henshall and Schuller 2012 [52]	Qualitative	Identify of low-value practices	Identification and prioritization approaches include clinical stakeholder involvement, monitoring new evidence, use of data to identify practices with high variability and/or cost, inclusion of HTR within life-cycle of any technology	No
Polisena et al. 2013 [19]	Systematic review	Facilitate the de-adoption process	Three different models to facilitate disinvestment decisions:	Yes; varied by included study
			1. Health technology assessment framework	
			2. Program budgeting and marginal analysis	
			3. Accountability for reasonableness and quality improvement theory	

*GuNFT* Guideline for Not Funding Health Technology, *HTR* health technology reassessment, *PBMA* program budgeting and marginal analysis

Lists of low-value practices were provided by eight citations (Table 5). Searches of the published literature were the most frequently employed means of identifying low-value practices (n = 7 citations, 88 %); however, the sources searched and the approach to defining a low-value practice varied by citation. Evidence was combined with stakeholder engagement to identify low-value practices in three citations [8, 21, 22], and one citation identified low-value practices as those shown to have high variability in rates of use between providers [23]. Among the seven citations that used the published literature to identify low-value practices, the prevalence of low-value practices ranged from 16 % [24] to 46 % [5], with two studies each identifying more than 100 low-value practices [7, 8].

**Table 5:** Original research citations that identified lists of low-value clinical practices

Citation	Stakeholder engagement	Single clinical area of focus	Methodology	Results
Ioannidis 2005 [24]	No	No	Broad literature search (1990–2003) for highly cited clinical research studies published in three major clinical journals^a^ or medical specialty journals with an impact factor >7.0	7 of 45 (16 %) highly cited studies claiming effectiveness eventually contradicted by replication research
			Supplemental, tailored searches to determine if each highly cited study had been replicated	7 other replication studies (16 %) found effect size not as large as in original study
			Comparison of direction of results between replicated and original highly cited study	
Prasad et al. 2011 [5]	No	No	Review of all “original articles” published in New England Journal of Medicine in 2009	35 of 124 (28 %) articles examined an existing medical practice
			Articles classified according to whether the practice examined was new or already in place, and whether the results were positive or negative for the primary endpoint	16 of 35 (46 %) articles examining an existing practice demonstrated medical reversal^b^
Elshaug et al. 2012 [8]	Comprehensive Management Framework for Australia’s Medicare Benefits Schedule	No	Environmental scanning approach triangulating data from broad PubMed search (2000–2010), targeted searches within select databases (e.g., Cochrane library), and opportunistic sampling among clinical and non-clinical stakeholders	156 potentially ineffective or unsafe practices identified from 5,209 screened articles
			Excluded pharmaceuticals	
Choosing Wisely 2012 [21]	Yes	Yes, specialty specific recommendations	Varied by specialty society but generally included one or more of literature search, expert opinion, and/or a modified Delphi process	67 specialty specific Top 5 ‘do not do’ lists
Garner et al. 2013 [70]	NICE	No	Present results from the first 6 months of the Cochrane Quality and Productivity project to identify low-value practices	28 of 65 (43 %) reviews published over a 6-month period identified potentially low-value practices
			Routine scanning of “implications for practice” section in new or updated Cochrane reviews to identify those wherein the author concluded an intervention is ineffective/harmful or should be confined to use within a research context	Most reviews cited a lack of randomized evidence of effectiveness, rather than robust evidence of lack of effectiveness
			Each review is examined to ensure it meets Cochrane Quality and Productivity criteria (potential impact on quality, safety, patient/provider experience, and potential for cash-releasing savings) for recommendation as a potential “disinvestment” candidate	To date the NICE Health Technology Appraisal Program has generated 1,347 ‘do not do’ recommendations [130]
Hollingworth et al. 2013 [23]	No	Yes, interventional procedures	Used UK Hospital Episode Statistics to identify inpatient interventional procedures with high variation in rates of use between PCTs in England	Substantial inter-procedure, inter-PCT variation in procedure rates
				Procedures with high variation not listed^c^
Prasad et al. 2013 [7]	No	No	Review of all original research articles published in New England Journal of Medicine from 2001 to 2010	363 of 1,344 (27 %) articles re-examined an established practice
			Articles classified according to whether the practice examined was new or already in place, and whether the results were positive or negative for the primary endpoint	146 of 363 (40 %) articles re-examining an existing practice demonstrated evidence of reversal
			Articles further classified as *replacement*, *back to the drawing board*, *reversal*, or *reaffirmation* ^d^	
Choosing Wisely Canada 2014 [22]	Yes	Yes, specialty specific recommendations	Varied by specialty society but generally included one or more of literature search, expert opinion, and/or a modified Delphi process	61 recommendations across 18 medical and surgical specialties

^a^Major clinical journals included *New England Journal of Medicine*, *Journal of the American Medical Association*, and *The Lancet*

^b^Medical reversal occurs when a new study—superior to predecessors because of better design, increased power, or more appropriate controls—contradicts current clinical practice [5]

^c^Conference abstract limited availability of data from this study

^d^Replacement = new practice surpasses older standard of care; back to the drawing board = new practice fails to surpass standard of care; reversal = current practice inferior to a lesser or prior standard; reaffirmation = existing practice superior to a lesser or prior standard

*NICE* National Institute for Health and Care Excellence, *PCT,* Primary Care Trusts

The impact of de-adoption efforts was evaluated and reported in 39 original research citations (Table 6). Most studies used interrupted time series methodology (n = 21, 54 %) and obtained data from large administrative databases or clinical registries (n = 30, 76 %). The most common target conditions were cardiovascular disease (n = 11, 28 %), arthritides (n = 8, 21 %), and menopause (n = 7, 18 %). All but one of the practices (pulmonary artery catheter) examined were therapeutic interventions. The most frequently examined therapies included cyclo-oxygenase-2 (COX-2) inhibitors and other non-steroidal anti-inflammatory drugs (NSAIDs) (n = 8, 21 %), hormone replacement therapy (n = 7, 18 %), and percutaneous coronary intervention (n = 3, 8 %). Thirteen studies reported on de-adoption efforts that followed an active change intervention, all of which demonstrated reductions in the target low-value practice [25–37]. The most common intervention was withdrawal of a low-value drug from the market (n = 9, 23 %). Other active change interventions commonly included an education component targeted at patients and/or providers. Of the 26 studies that did not report on the effects of an active change intervention, 23 (88 %) demonstrated reductions in the target practice. Of the 27 and 11 studies that examined de-adoption efforts for harmful or ineffective practices, respectively, 25 (92 %) and 9 (81 %) demonstrated reductions in the target practice.

**Table 6:** Original research citations that evaluated the de-adoption of low-value clinical practices^a^

Citation	Study design	Target condition	Low-value practice	Evidence guiding de-adoption	Reason practice considered low-value	Reduction in use of low-value practice	Other notable results
*Active change intervention facilitated de-adoption* ^*b*^							
Ross-Degnan et al. 1993 [29]	Interrupted time series	Arthritides	NSAIDs, Zomepirac	Case series	Harmful	Yes	Increased prescription of other NSAIDs
Williams et al. 2006 [30]	Interrupted time series	Arthritides	COX-2 inhibitors	RCT	Harmful	Yes	Safety concerns for rofecoxib interpreted as class effect
Thiebaud et al. 2006 [31]	Cohort study	Arthritides	COX-2 inhibitors	RCT	Harmful	Yes	Greater decrease in COX-2 inhibitor use among patients with greater number of cardiovascular comorbidities
Barozzi and Tett 2007 [32]	Interrupted time series	Arthritides	COX-2 inhibitors	RCT	Harmful	Yes	Safety concerns for rofecoxib interpreted as class effect; prescription of non-selective NSAIDs increased
Sun et al. 2007 [33]	Interrupted time series	Arthritides	COX-2 inhibitors	RCT	Harmful	Yes	Significant increases in non-selective NSAID use after withdrawal of rofecoxib and valdecoxib
Setakis et al. 2008 [34]	Before-and-after	Arthritides	COX-2 inhibitors	RCT	Harmful	Yes	After withdrawal of rofecoxib, remaining use of COX-2 inhibitors did not concentrate in patients with high gastrointestinal risk and low cardiovascular risk
Sukel et al. 2008 [35]	Before-and-after	Arthritides	COX-2 inhibitors	RCT	Harmful	Yes	Safety concerns for rofecoxib interpreted as class effect
Hsiao et al. 2009 [36]	Cohort	Arthritides	COX-2 inhibitors	RCT	Harmful	Yes	Safety concerns for rofecoxib interpreted as class effect
Stafford and Radley 2003 [37]	Interrupted time series	Obesity	Fenfluramine and dexfenfluramine	Case–control study	Harmful	Yes	No change in practice after reports of adverse events. Market withdrawal of drug required to change practice
Krol et al. 2004 [27]	Cluster RCT	PPI use	PPIs in those without indications for their continued use	Clinical practice guideline	Not reported	Yes	No recrudescence of symptomatology associated with original PPI prescription after its discontinuation
Roumie et al. 2004 [25]	Interrupted time series	Post-menopausal women	HRT	RCT	Harmful	Yes	Greater rate of discontinuation of HRT after tailored de-adoption intervention compared to media release of results of WHI study
Kulawik et al. 2009 [28]	Before-and-after	End-stage renal disease	Use of tunnelled hemodialysis catheters in patients with end-stage renal disease	Cohort, quasi-experimental, and clinical practice guideline	Harmful, not cost effective	Yes	Involvement of medical leader improved rate of reduction in catheter use
Sindby et al. 2011 [26]	Before-and-after	Coronary artery bypass surgery	Blood transfusions	Not reported	Not reported	Yes	Not reported (conference abstract)
*No intervention used to facilitate de-adoption* ^*c*^							
Austin et al. 2003 [99]	Interrupted time series	Post menopausal women	HRT	RCT	Harmful	Yes	Unable to determine if decline in HRT use patient or physician-initiated
Lawton et al. 2003 [124]	Survey	Post menopausal women	HRT	RCT	Harmful	Yes	Factors associated with stopping HRT included older age, use of combined HRT, longer duration of HRT
Haas et al. 2004 [118]	Interrupted time series	Post menopausal women	HRT	RCT	Harmful	Yes	Greater decrease in HRT use after WHI study compared to Heart and Estrogen/progestin Replacement Study
Hersh et al. 2004 [103]	Interrupted time series	Post menopausal women	HRT	RCT	Harmful	Yes	Response to publication of WHI study was rapid
Majumdar et al. 2004 [98]	Interrupted time series	Post menopausal women	HRT	RCT	Harmful	Yes	Substantial decline in promotional spending for HRT after publication of WHI study
Huang et al. 2007 [122]	Cohort	Post menopausal women	HRT	RCT	Harmful	Yes	Factors associated with reduction in use of HRT included higher patient education, and care at an academic institution
Majumdar et al. 2001 [97]	Before-and-after	Acute coronary syndrome	Calcium channel blockers Lidocaine	Case–control study; Systematic review	Harmful	Yes	No difference in calcium channel blocker discontinuation according to physician specialty
Brunt et al. 2003 [120]	Interrupted time series	Hypertension	Short acting calcium channel blockers	Case–control study	Harmful	Yes	Proportionate increase in other anti-hypertensive medication paralleled discontinuation of calcium channel blockers
Stafford et al. 2004 [96]	Interrupted time series	Hypertension	Alpha-blockers	RCT	Harmful	Yes	Substantial decrease in office promotion expenditures for alpha-blockers following publication of ALLHAT trial
Xie et al. 2005 [95]	Interrupted time series	Hypertension	Alpha-blockers	RCT	Harmful	Yes	Decrease in alpha-blockers associated with increase in other anti-hypertensive medications
Hauptman et al. 2006 [18]	Interrupted time series	Congestive heart failure	Nesiritide	Systematic review	Harmful	Yes	Decrease in nesiritide use associated with increased use of inotropes
Atwater et al. 2009 [90]	Before-and-after	Coronary artery disease	PCI	RCT	Lack of efficacy	Yes	Decrease in PCI and increase in medical therapy following COURAGE trial
Bonakdar tehrani and Howard 2011 [84]	Before-and-after	Coronary artery disease	PCI	RCT	Lack of efficacy	Yes	PCI use decreased after COURAGE trial, however considerable number of patients with stable angina continued to receive PCI
Deyell et al. 2011 [89]	Interrupted time series	Coronary artery disease	PCI	RCT	Lack of efficacy	No	No change in PCI after OAT trial or guideline revisions
Ahmed et al. 2011 [93]	Interrupted time series	Coronary artery disease	PCI	RCT	Lack of efficacy	Yes	Decrease in PCI use was sustained up to 2 years after publication of COURAGE trial
Wiener and Welch 2007 [91]	Interrupted time series	Critical illness	PAC	RCT; Systematic review	Lack of efficacy	Yes	PAC use began to decline after publication of large observational study (before publication of any RCTs)
Koo et al. 2011 [87]	Interrupted time series	Critical illness	PAC	RCT; Systematic review	Lack of efficacy	Yes	Examined patient, physician, and unit-level predictors of PAC use
Gershengorn and Wunsch 2013 [128]	Cohort	Critical illness	PAC	RCT; Systematic review	Lack of efficacy	Yes	Surgical patients continue to have high likelihood of PAC use
Murphy et al. 2013 [92]	Cohort	Critical illness	Blood transfusions	RCT	Harmful	Yes (higher volume hospitals only)	Likelihood of receiving blood transfusion after publication of TRICC trial dependent on annualized intensive care unit patient volume
Duffy and Farley 1992 [105]	Cohort	Chronic obstructive pulmonary disease	IPPB	RCT	Lack of efficacy	Yes	Hospital-level traits and models of funding technologies were associated with discontinuing IPPB
Smalley et al. 2000 [121]	Before-and-after	Gastric motility disorders	Cisapride	Case series; Warning letter from Food and Drug Administration	Harmful	No	Cisapride use not effected by black-box US Food and Drug Administration warning regarding harmful effects
Howard et al. 2011 [101]	Interrupted time series	Breast cancer	High dose chemotherapy/Hematopoietic cell transplants	RCT	Lack of efficacy and harmful	Yes	No association between hospital teaching status and participation in clinical trials, and decline in use of the low-value practice
Chamberlain et al. 2013 [56]	Interrupted time series	(1) Pregnant women with hepatitis	(1) Caesarean section	Clinical practice guideline (NICE ‘do not do’ recommendation)	Lack of efficacy and harmful	No	“Do not do” recommendation reminders had no association with changes in clinical practice
		(2) Infertile men and women	(2) Fertility procedures				
Kowalczyk et al. 2012 [82]	Cohort	Prostate cancer	RRP	Cohort study	Not reported	Yes	Decrease in RRP was associated with an increase in RRP-related complications
Luetmer and Kallmes 2011 [88]	Before-and-after	Vertebral fracture	Vertebroplasty	RCT	Lack of efficacy	Yes	Referrals for vertebroplasty decreased, however proportion of referrals undergoing the procedure increased
Ehrenstein et al. 2013 [94]	Interrupted time series	Diabetes mellitus	Rosiglitazone	Systematic review Cohort study	Harmful	Yes	No significant change in markers of glycemic control after discontinuation of rosiglitazone

^a^Five citations excluded from this table discussed, but did not actually evaluate the outcome of a de-adoption process [52, 59, 106, 123, 125]

^b^Citations that employed a de-adoption intervention included:

- Ross-Degnan et al. [29]: Market withdrawal of Zomepirac

- Williams et al. [30], Thiebud et al. [31], Barozzi and Tett [32], Sun et al. [33], Setakis et al. [34], Sukel et al. [35], Hsiao et al. [36]: Market withdrawal of rofecoxib

- Stafford and Radley [37]: Market withdrawal of fenfluramine and dexfenfluramine

- Krol et al. [27]: Information leaflet with recommendations for reducing inappropriate PPI use sent to patients from general practice clinics

- Roumie et al. [25]: Three-part intervention consisting of patient and provider education component and provider care component

- Kulawik et al.,[28]: Catheter reduction toolkit (education on types of vascular access) employed in facilities with high catheter utilization rates

- Sindby et al. [26]: Provider education, audit and feedback, and hospital-level guideline changes

^c^Any observed de-adoption reflects the effect of passive diffusion of evidence of a practice’s ineffectiveness or harm

*COX-2* cyclo-oxygenase-2, *HRT* hormone replacement therapy, *IPPB* intermittent positive pressure breathing, *NSAIDs* non-steroidal anti-inflammatory drugs, *PAC* pulmonary artery catheter, *PCI* percutaneous coronary intervention, *PPIs* proton pump inhibitors, *RRP* retropubic radical prostatectomy, *WHI* Women’s Health Initiative

## Discussion

**Fig. 4:**
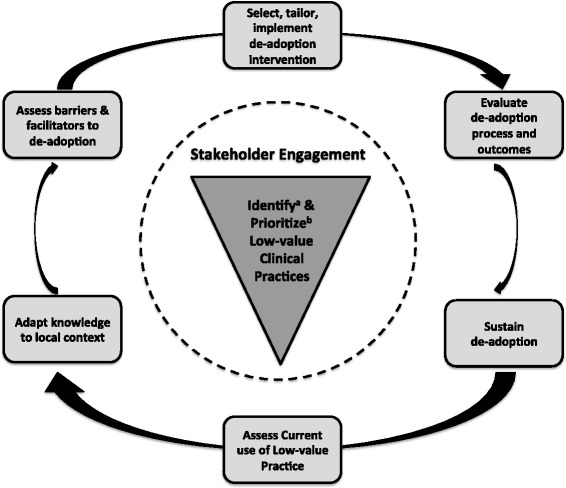
Synthesis model for the process of de-adoption. ^a^ Identification of low-value practices includes the process of reviewing and selecting de-adoption knowledge. ^b^ Current literature suggests prioritizing based on safety of the low-value practice (i.e., harmful practices eliminated first), potential health and cost impact of de-adoption, and availability of alternative practices

De-adoption of low-value clinical practices is essential to improve healthcare quality and create a sustainable healthcare system. To our knowledge this is the first knowledge synthesis to comprehensively examine the de-adoption of low-value clinical practices. We identified 109 citations, most of which were published within the last five years, and concentrated on evaluating changes in practice that occurred following the publication of evidence for medical reversal. We identified 43 terms used to refer to the process of de-adoption, with *disinvest* being the most frequently cited term. We also identified 13 frameworks that conceptualize individual components of the de-adoption process, and from these frameworks propose a model for de-adoption (Fig. 4). These results provide foundations for guiding the de-adoption of ineffective and harmful clinical practices from patient care as well as directing future research.

The first major finding from our study pertains to the diverse list of terms used to refer to de-adoption with no clearly established taxonomy. The implication of this is that communication is impaired, which may impact “branding” of de-adoption and efficient searching for relevant literature. Furthermore, it is unclear how different concepts and initiatives such as “less is more” [38], reducing research waste [39], and Choosing Wisely [40] are related. Conversely, knowledge translation and implementation science are increasingly recognized terms in healthcare research, facilitating understanding and communication of the related concepts. Terms such as de-adoption and de-implementation that have a more general connotation, and are natural antonyms of adoption and implementation, ought to be used as terms that brand the process of reducing or removing low-value clinical practices. Other terms, such as disinvest, describe specific elements of the de-adoption process and are not ideal candidates to brand this process. Interestingly, de-adoption and de-implementation were infrequently cited within the included citations, whereas disinvest was the most commonly cited term. Given this lack of clarity with regard to de-adoption terminology, there is an urgent need to develop a taxonomy of terms.

Using the proposed conceptual framework (Table 1), themes common to the frameworks identified in the scoping review (Table 4), and the Knowledge-to-Action framework [41], we derived the second major result from this study, a synthesis framework for facilitating de-adoption (Fig. 4). At the heart of this framework is the identification and prioritization of low-value practices. The identification process involves determining the low-value practice(s) and selection of the knowledge unit that defines a practice as low-value (i.e., randomized clinical trial, systematic review, and/or clinical practice guideline). With regard to prioritization when there is more than one low-value practice identified, current literature suggests prioritizing based on strength of evidence supporting lack of efficacy, safety of the low-value practice (i.e., harmful practices eliminated first), potential health and cost impact of de-adoption, and availability of alternative practices. To permit more of an integrated de-adoption process, and thus improve the probability of success, we suggest stakeholder engagement take place concomitant with practice identification and prioritization. The de-adoption process is then envisioned to follow a similar action cycle as in the original Knowledge-to-Action cycle [41]. However, given the anticipated challenges associated with discontinuing established clinical practices [42], the analysis of barriers and facilitators will require a greater in-depth exploration of both scientific (e.g., presence and quality of evidence supporting de-adoption) and non-scientific (e.g., historical, political, social, and economic factors) barriers to de-adoption [43]. In addition, the intervention that guides de-adoption will likely need to be more closely integrated into clinical care pathways compared to that for adoption, with policy changes and/or changes to funding models predicted to have the greatest likelihood of facilitating de-adoption. Implementation of the intervention will need to be evaluated, and outcomes such as low-value practice use, costs, and potential harms assessed. Finally, any de-adoption intervention should include a sustainability plan; else it is highly likely that healthcare providers will (knowingly or unknowingly) revert to using the practice to which they have become habituated [44].

The third important result from this review is the identification of key questions that require additional research to advance the science of de-adoption. For example, there are multiple factors that likely determine when a practice should be de-adopted (e.g., nature of the intervention, lack of effectiveness or degree of harm, nature of the evidence) but the role of each factor and the interplay among them that ultimately determines when to de-adopt is not clear. In addition, what do we do with clinical practices that are ineffective for a broad population, but may be effective in a small subgroup that is difficult to study? To answer these and other questions we need additional knowledge syntheses that establish a taxonomy of de-adoption terminology, summarize barriers and facilitators to de-adoption, and quantify the impact of past examples of de-adoption. We also need empirical research to examine optimal strategies for identifying candidate low-value practices, and to determine which de-adoption strategies are likely to have the greatest impact. Furthermore, given existing fiscal climates with limited resources, we also need to balance the need to refine and prioritize the science of de-adoption with the need to do the same for adopting new practices.

While we await this additional research, what can healthcare decision-makers practically do with the existing knowledge base? First, this review highlights that de-adoption requires a multi-dimensional construct that is far more complex than simply ceasing to provide a given practice. Second, several studies have demonstrated that de-adoption does occur in response to publication of new evidence (Table 6), with the most consistent de-adoption occurring in response to an active change intervention. The intervention with the greatest likelihood of de-adoption is market withdrawal of a harmful drug. However, the real challenge lies in how to actively facilitate de-adoption when market withdrawal is not possible (e.g., insulin [45]), or not clearly indicated (e.g., practices that are simply ineffective). Interventions cited as having the greatest likelihood of effecting de-adoption include changes to policies, and/or restructuring of funding associated with the low-value practice, the latter through strategies of disinvestment, reinvestment, or defunding. However, this scoping review did not identify any studies that applied a strategy of disinvestment in response to evidence for medical reversal. At this point, pending further research, we suggest use of our proposed synthesis model (Fig. 4) as a starting point for anyone interested in promoting the de-adoption of low-value practices.

There are limitations to this review. First, our search may have missed relevant articles due to the lack of indexing terminology specific to de-adoption that for practical reasons forced us to restrict the search to English language articles published from 1990 onwards. However, the majority of included citations were published after 1999, and originated in high-income countries, therefore it is unlikely that we missed any broad concepts related to de-adoption. Second, grouping articles and de-adoption terminology according to the main categories in the conceptual framework, even though completed in duplicate by independent reviewers, is partly subjective. Finally, we elected to conduct a scoping review in order to provide an inclusive and broad description of what is known about de-adoption and therefore are limited in our ability to present granular details. Our work identifies opportunity for future systematic reviews.

## Conclusions

De-adoption of low-value clinical practices is essential to improve healthcare quality and create a sustainable healthcare system. We identified a large body of literature that describes current approaches, and challenges to the de-adoption of low-value clinical practices. Our results should promote future research in at least two areas. First, knowledge syntheses are required to explore areas wherein there is an abundance of literature, such as establishing a taxonomy of de-adoption terminology, summarizing barriers and facilitators to de-adoption, and quantifying the impact of past examples of de-adoption. Second, empirical research is required to examine optimal strategies for identifying candidate low-value practices, and to determine which de-adoption strategies are likely to have the greatest impact. In the meantime, we have developed a conceptual model that providers and decision-makers can use to guide efforts to de-adopt ineffective and harmful practices and describe examples of successful de-adoption that can be used to inform efforts.

## Additional file

Additional file 1:
**Table S1.** Description of articles included in the scoping review. Appendix. MEDLINE search. (DOCX 201 kb)

## Abbreviations

CADTH: Canadian Agency for Drugs and Technologies in Health
CENTRAL: Cochrane Central Register of Controlled Trials
COX-2: cyclo-oxygenase-2
GuNFT: Guideline for Not Funding Health Technology
HERS: Heart and Estrogen/progestin Replacement Study
HRT: hormone replacement therapy
HTR: health technology reassessment
IPPB: intermittent positive pressure breathing
IQR: inter-quartile range
MeSH: Medical Subject Heading
NICE: National Institute for Health and Care Excellence
NSAIDs: non-steroidal anti-inflammatory drugs
PAC: pulmonary artery catheter
PBMA: program budgeting and marginal analysis
PCI: percutaneous coronary intervention
PCT: Primary Care Trusts
PPI: proton pump inhibitor
PRESS: Peer Review of Electronic Search Strategies
PRISMA: Preferred Reporting Items for Systematic Reviews and Meta-analyses
RCT: randomized controlled trial
RRP: retropubic radical prostatectomy
STEMI: ST-elevation myocardial infarction
WHI: Women’s Health Initiative
