# Supplementary material for: Towards understanding the de-adoption of low-value clinical practices: a scoping review
Source: BMC Med. 2015 Oct 6;13:255. doi: 10.1186/s12916-015-0488-z (PMC4596285; doi:10.1186/s12916-015-0488-z)
Supplement: Additional file 1: — Table S1. Description of articles included in the scoping review. Appendix. MEDLINE search. (DOCX 201 kb) [file 12916_2015_488_MOESM1_ESM.docx]

**Supplementary Online Material**

Niven DJ, Mrklas KJ, Holodinsky JK, Straus SE, Hemmelgarn BR, Jeffs LP, Stelfox HT. Towards Understanding the De-adoption of Low-value Clinical Practices: A Scoping Review

**Table S1.** Description of articles included in the scoping review.

**Appendix.** MEDLINE search

**References**

**Table S1.** Description of articles included in the scoping review.

|  |  | **Focus of Article** | | | |  |
| --- | --- | --- | --- | --- | --- | --- |
| **Source** | **Article Type** | **Identify Low-value Practices** | **Facilitate De-adoption Process** | **Evaluate De-adoption Outcome** | **Sustain De-adoption** | **Cited De-adoption Barrier/ Facilitator** |
| Ahmed et al., 2011 [1] | Interrupted time series analysis | No | No | Yes | Yes | No |
| Atkins, 2009 [2] | Editorial | No | Yes | No | No | No |
| Atwater et al., 2009 [3] | Before-and-after study | No | No | Yes | No | Yes |
| Austin et al, 2003 [4] | Interrupted time series analysis | No | No | Yes | No | No |
| Azermai et al., 2014 [5] | Mixed methods | No | Yes | No | No | Yes |
| Banta and Thacker, 1990 [6] | Narrative review | Yes | Yes | No | No | Yes |
| Barozzi and Tett, 2007 [7] | Interrupted time series analysis | No | No | Yes | No | No |
| Bonakdar tehrani and Howard, 2011 [8] | Before-and-after study | No | No | Yes | No | No |
| Brunt et al, 2003 [9] | Interrupted time series analysis | No | No | Yes | No | Yes |
| Chamberlain et al., 2013 [10] | Interrupted time series analysis | No | No | Yes | No | No |
| Choosing Wisely, 2012 [11] | Summary of specialty society recommendations | Yes | No | No | No | No |
| Choosing Wisely Canada, 2014 [12] | Summary of specialty society recommendations | Yes | Yes | No | No | No |
| Colmenares, 2012 [13] | Narrative review | Yes | No | No | No | No |
| Cooper and Starkey, 2010 [14] | Editorial | Yes | Yes | Yes | No | Yes |
| Deyell et al., 2011 [15] | Interrupted time series analysis | No | No | Yes | No | Yes |
| Donaldson et al., 2010 [16] | Narrative review | Yes | Yes | No | No | Yes |
| Duffy and Farley, 1992 [17] | Cohort study | No | No | Yes | No | No |
| Ehrenstein et al., 2013 [18] | Interrupted time series analysis | No | No | Yes | No | No |
| Elshaug et al., 2007 [19] | Narrative review | Yes | Yes | Yes | No | Yes |
| Elshaug et al., 2008 [20] | Qualitative | No | Yes | No | No | Yes |
| Elshaug et al., 2009 [21] | Narrative review | Yes | No | No | No | Yes |
| Elshaug et al., 2009 [22] | Discussion paper | Yes | Yes | No | No | Yes |
|  |  | **Focus of Article** | | | |  |
| **Source** | **Article Type** | **Identify Low-value Practices** | **Facilitate De-adoption Process** | **Evaluate De-adoption Outcome** | **Sustain De-adoption** | **Cited De-adoption Barrier/ Facilitator** |
| Elshaug et al., 2012 [23] | Mixed methods | Yes | No | No | No | No |
| Elshaug et al., 2013 [24] | Editorial | Yes | No | No | No | Yes |
| Fatovich, 2013 [25] | Narrative review | Yes | No | No | No | No |
| Garner and Littlejohns., 2011 [26] | Editorial | Yes | Yes | Yes | No | Yes |
| Garner, 2012 [27] | Presentation on low-value care | Yes | Yes | Yes | No | Yes |
| Garner et al., 2013 [28] | Cohort study of articles | Yes | No | No | No | No |
| Gerdvilaite and Nachtnebel, 2011 [29] | Systematic review | No | Yes | No | No | Yes |
| Gershengorn and Wunsch, 2013 [30] | Cohort study | No | No | Yes | No | Yes |
| Haas et al., 2004 [31] | Interrupted time series analysis | No | No | Yes | No | Yes |
| Haas et al., 2012 [32] | Narrative review | Yes | Yes | No | No | Yes |
| Haines et al., 2014 [33] | Narrative review | No | Yes | Yes | No | No |
| Harris et al., 2013 [34] | Workshop synopsis | Yes | Yes | Yes | No | Yes |
| Hauptman et al., 2006 [35] | Interrupted time series analysis | No | No | Yes | No | Yes |
| Henshall et al., 2012 [36] | Qualitative | Yes | Yes | Yes | No | Yes |
| Hersh et al, 2004 [37] | Interrupted time series analysis | No | No | Yes | No | Yes |
| Hislop, 2011 [38] | Qualitative | Yes | Yes | No | No | Yes |
| Hodgetts et al., 2012 [39] | Mixed methods | Yes | Yes | No | No | Yes |
| Hollingsworth et al., 2013 [40] | Cohort study | Yes | No | No | No | No |
| Howard et al., 2011 [41] | Interrupted time series analysis | No | No | Yes | No | Yes |
| Howard et al., 2012 [42] | Predictive modelling | No | Yes | Yes | No | Yes |
| Hsiao et al, 2009 [43] | Cohort study | No | No | Yes | No | Yes |
| Huang et al., 2007 [44] | Cohort study | No | No | Yes | No | Yes |
| Hughes and Ferner, 2010 [45] | Editorial | Yes | No | No | No | Yes |
| Ibargoyen-Roteta et al., 2009 [46] | Survey | Yes | No | No | No | Yes |
|  |  |  |  |  |  |  |
|  |  | **Focus of Article** | | | |  |
| **Source** | **Article Type** | **Identify Low-value Practices** | **Facilitate De-adoption Process** | **Evaluate De-adoption Outcome** | **Sustain De-adoption** | **Cited De-adoption Barrier/ Facilitator** |
| Ibargoyen-Roteta et al., 2010 [47] | Guideline | Yes | Yes | Yes | No | Yes |
| Ioannidis, 2013 [48] | Letter-to-the-editor | Yes | No | No | No | Yes |
| Ioannidis, 2005 [49] | Cohort study of articles | Yes | No | No | No | Yes |
| Joshi et al., 2009 [50] | Discussion paper | Yes | Yes | No | No | Yes |
| Karnon et al., 2009 [51] | Predictive modelling | Yes | No | No | No | No |
| Kelly et al., 2006 [52] | Commissioned report | Yes | Yes | No | No | Yes |
| Kiechle et al., 2014 [53] | Narrative review | Yes | Yes | No | No | Yes |
| Koo et al., 2011 [54] | Interrupted time series analysis | No | No | Yes | No | Yes |
| Kowalczyk et al., 2012 [55] | Cohort study | No | No | Yes | No | No |
| Krol et al., 2004 [56] | Randomized clinical trial | No | Yes | Yes | No | Yes |
| Kulawik et al., 2009 [57] | Before-and-after study | No | Yes | Yes | No | Yes |
| Lawton et al., 2003 [58] | Survey | No | No | Yes | No | Yes |
| Lifeinthefastlane.com [59] | Website | Yes | No | No | No | No |
| Leggett et al., 2012 [60] | Mixed methods | Yes | Yes | Yes | No | Yes |
| Leggett et al., 2012 [61] | Systematic review | Yes | Yes | No | No | No |
| Leng, 2006 [62] | Government project plan | Yes | Yes | No | No | No |
| Levin, 2011 [63] | Presentation of Ontario disinvestment initiatives | No | No | Yes | No | Yes |
| Luetmer and Kallmes, 2011 [64] | Before-and-after study | No | No | Yes | No | No |
| MacKean et al., 2013 [65] | Consensus panel report | Yes | Yes | No | No | Yes |
| Majumdar et al., 2001 [66] | Before-and-after study | No | No | Yes | No | No |
| Majumdar et al., 2004 [67] | Interrupted time series analysis | No | No | Yes | No | Yes |
| Massatti et al., 2008 [68] | Mixed methods | No | Yes | No | Yes | Yes |
| Mortimer, 2010 [69] | Narrative review | Yes | Yes | No | No | Yes |
| Mosucci, 2011 [70] | Editorial | Yes | Yes | No | No | Yes |
| Moynihan, 2012 [71] | Editorial | Yes | Yes | Yes | No | Yes |
| Murphy et al., 2013 [72] | Cohort study | No | No | Yes | No | Yes |
| Naylor, 2004 [73] | Editorial | No | Yes | Yes | No | Yes |
| Nieuwlatt et al., 2013 [74] | Narrative review | No | Yes | No | No | No |
| Noseworthy and Clement, 2012 [75] | Editorial | No | Yes | No | No | Yes |
| Nuti et al., 2010 [76] | Predictive modelling | Yes | No | No | No | No |
|  |  | **Focus of Article** | | | |  |
| **Source** | **Article Type** | **Identify Low-value Practices** | **Facilitate De-adoption Process** | **Evaluate De-adoption Outcome** | **Sustain De-adoption** | **Cited De-adoption Barrier/ Facilitator** |
| Paulden, 2012 [77] | Predictive modelling | Yes | No | No | No | No |
| Pearson and Littlejohns, 2007 [78] | Narrative review | Yes | Yes | Yes | No | Yes |
| Polisena et al., 2013 [79] | Systematic review | Yes | No | No | No | Yes |
| Prasad et al., 2011 [80] | Cohort study of articles | Yes | No | No | No | No |
| Prasad et al., 2011 [81] | Narrative review | Yes | No | No | No | No |
| Prasad et al., 2012 [82] | Editorial | Yes | No | No | No | No |
| Prasad and Vandross, 2012 [83] | Narrative review | Yes | No | No | No | No |
| Prasad et al., 2013 [84] | Cohort study of articles | Yes | No | No | No | No |
| Prasad and Cifu, 2013 [85] | Letter-to-the-editor (response) | Yes | No | No | No | No |
| Prasad and Ioannidis, 2014 [86] | Narrative review | Yes | No | No | No | Yes |
| Prescrire International, 2013 [87] | Narrative review | Yes | No | No | No | No |
| Ross-Degnan et al., 1993 [88] | Interrupted time series analysis | No | No | Yes | No | No |
| Roumie et al., 2004 [89] | Interrupted time series | No | No | Yes | No | No |
| Setakis et al., 2008 [90] | Before-and-after study | No | No | Yes | No | No |
| Sindby et al., 2011 [91] | Before-and-after study | No | No | Yes | No | No |
| Siontis et al., 2009 [92] | Cohort study of articles | No | Yes | No | No | Yes |
| Smalley et al., 2000 [93] | Before-and-after study | No | No | Yes | No | No |
| Stafford and Radley, 2003 [94] | Interrupted time series analysis | No | No | Yes | No | No |
| Stafford et al., 2004 [95] | Interrupted time series analysis | No | No | Yes | No | Yes |
| Sukel et al., 2008 [96] | Before-and-after study | No | No | Yes | No | Yes |
| Sun et al., 2007 [97] | Interrupted time series analysis | No | No | Yes | No | No |
| Street et al., 2011 [98] | Qualitative | No | Yes | No | No | Yes |
| Tatsioni et al., 2007 [99] | Cohort study of articles | No | Yes | No | No | Yes |
| Tatsioni et al., 2010 [100] | Cohort study of articles | No | Yes | No | No | Yes |
| Thiebaud et al., 2006 [101] | Cohort study | No | No | Yes | No | Yes |
| Voorn et al., 2012 [102] | Mixed methods | No | No | Yes | No | Yes |
| Warner et al., 2013 [103] | Editorial | Yes | No | No | No | No |
|  |  | **Focus of Article** | | | |  |
| **Source** | **Article Type** | **Identify Low-value Practices** | **Facilitate De-adoption Process** | **Evaluate De-adoption Outcome** | **Sustain De-adoption** | **Cited De-adoption Barrier/ Facilitator** |
| Watt et al., 2012 [104] | Mixed methods | No | Yes | No | No | Yes |
| Watt et al., 2012 [105] | Stakeholder engagement exercise | No | Yes | No | No | Yes |
| Wiener and Welch, 2007 [106] | Interrupted time series analysis | No | No | Yes | No | No |
| Williams et al., 2006 [107] | Interrupted time series analysis | No | No | Yes | No | No |
| Williams and Bryan, 2007 [108] | Mixed methods | No | Yes | No | No | No |
| Xie et al., 2005 [109] | Interrupted time series analysis | No | No | Yes | No | No |

**Appendix – MEDLINE Search (March 5, 2014)**

1. ((abandon* or contradict* or refute* or refuting or reassess* or re-assess* or obsole* or revers* or delist* or de-list* or disinvest* or dis-invest* or discontinu* or dis-continu* or decommission* or de-commission* or deadopt* or de-adopt* or de-implement* or deimplement* or "health care' withdraw*" or (no adj benefit*)) adj5 (healthcare or technolog* or device* or intervention* or health practi?e* or medical or medical practi?e* or procedur* or drug or drugs or biotechnology*)).tw.
2. limit 1 to English language
3. limit 2 to animals
4. limit 2 to (animals and humans)
5. 3 not 4
6. 2 not 5
7. limit 6 to (“all infant (birth to 23 months)” or “newborn infant (birth to 1 month)” or “infant (1 to 23 months)” or “preschool child (2 to 5 years)” or “child (6 to 12 years)”)
8. 6 not 7
9. Limit 8 to yr=”1990-Current”

REFERENCES

1. Ahmed B, Dauerman HL, Piper WD, Robb JF, Verlee MP, Ryan TJ, Jr., Goldberg D, Boss RA, Jr., Phillips WJ, Fedele F *et al*: **Recent changes in practice of elective percutaneous coronary intervention for stable angina**. *Circulation Cardiovasc Qual Outcomes* 2011, **4**:300-305.

2. Atkins D: **Connecting Research and Patient Care: Lessons from the VA’s Quality Enhancement Research Initiative**. *J Gen Intern Med* 2009, **25**:1-2.

3. Atwater BD, Oujiri J, Wolff MR: **The immediate impact of the Clinical Outcomes Utilizing Revascularization and Aggressive Drug Evaluation (COURAGE) trial on the management of stable angina.** *Clin Cardiol* 2009, **32**:E1-E3.

4. Austin PC, Mamdani MM, Tu K, Jaakkimainen L: **Prescriptions for estrogen replacement therapy in Ontario before and after publication of the Women's Health Initiative Study**. *JAMA* 2003, **289**:3241-3242.

5. Azermai M, Vander Stichele RR, Van Bortel LM, Elseviers MM: **Barriers to antipsychotic discontinuation in nursing homes: an exploratory study**. *Aging Ment Health* 2014, **18**:346-353.

6. Banta HD, Thacker SB: **The case for reassessment of health care technology. Once is not enough**. *JAMA* 1990, **264**:235-240.

7. Barozzi N, Tett SE: **What happened to the prescribing of other COX-2 inhibitors, paracetamol and non-steroidal anti-inflammatory drugs when rofecoxib was withdrawn in Australia?** *Pharmacoepidemiol Drug Saf* 2007, **16**:1184-1191.

8. Howard D: **Potential cost savings from comparative effectiveness research: Lessons from courage study Bonakdar tehrani a**. *Value in Health* 2011, **14 (3)**:A2.

9. Brunt ME, Murray MD, Hui SL, Kesterson J, Perkins AJ, Tierney WM: **Mass media release of medical research results: an analysis of antihypertensive drug prescribing in the aftermath of the calcium channel blocker scare of March 1995**. *J Gen Intern Med* 2003, **18**:84-94.

10. Chamberlain CA, Martin RM, Busby J, Gilbert R, Cahill DJ, Hollingworth W: **Trends in procedures for infertility and caesarean sections: was NICE disinvestment guidance implemented? NICE recommendation reminders**. *BMC Public Health* 2013, **13**:112.

11. **Choosing Wisely: Five Things Physicians and Patients Should Question**. American Board of Internal Medicine Foundation. 2012. http://www.choosingsiwely.org. Accessed 4 Mar 2014

12. **Choosing Wisely Canada**. Canadian Medical Association. 2014. http://www.choosingwiselycanada.org. Accessed August 8, 2014

13. Colmenares P: **Proposal for a state health technology assessment program**. *WMJ* 2012, **111**:176-182.

14. Cooper C, Starkey K: **Disinvestment in health care**. *BMJ* 2010, **340**:c1413-c1413.

15. Deyell MW, Buller CE, Miller LH, Wang TY, Dai D, Lamas GA, Srinivas VS, Hochman JS: **Impact of National Clinical Guideline recommendations for revascularization of persistently occluded infarct-related arteries on clinical practice in the United States**. *Arch Int Med* 2011, **171**:1636-1643.

16. Donaldson C, Bate A, Mitton C, Dionne F, Ruta D: **Rational disinvestment**. *QJM* 2010, **103**:801-807.

17. Duffy SQ, Farley DE: **The protracted demise of medical technology. The case of intermittent positive pressure breathing**. *Med Care* 1992, **30**:718-736.

18. Ehrenstein V, Hernandez RK, Ulrichsen SP, Rungby J, Lash TL, Riis AH, Li L, Sorensen HT, Jick SS: **Rosiglitazone use and post-discontinuation glycaemic control in two European countries, 2000-2010**. *BMJ Open* 2013, **3**:e003424.

19. Elshaug AG, Hiller JE, Tunis SR, Moss JR: **Challenges in Australian policy processes for disinvestment from existing, ineffective health care practices**. *Aust New Zealand Health Policy* 2007, **4**:23.

20. Elshaug AG, Hiller JE, Moss JR: **Exploring policy-makers' perspectives on disinvestment from ineffective healthcare practices**. *Int J Technol Assess Health Care* 2008, **24**:1-9.

21. Elshaug AG, Moss JR, Littlejohns P, Karnon J, Merlin TL, Hiller JE: **Identifying existing health care services that do not provide value for money**. *Med J Austr* 2009, **190**:269-273.

22. Elshaug AG, Watt AM, Moss JR, Hiller JE: **Policy Perspectives on the Obsolescence of Health Technologies in Canada**. Canadian Agency for Drugs and Technologies in Health. 2009. https://www.cadth.ca/media/pdf/Obsolescence%20of%20Health%20Technologies%20in%20Canada_Policy_Forum_e.pdf. Accessed 21 Oct 2013.

23. Elshaug AG, Watt AM, Mundy L, Willis CD: **Over 150 potentially low-value health care practices: an Australian study**. *Med J Austr* 2012, **197**:556-560.

24. Elshaug AG, McWilliams JM, Landon BE: **The value of low-value lists**. *JAMA* 2013, **309**:775-776.

25. Fatovich DM: **Medical reversal: What are you doing wrong for your patient today?** *Emerg Med Australas* 2013, **25**:1-3.

26. Garner S, Littlejohns P: **Disinvestment from low value clinical interventions: NICEly done?** *BMJ* 2011, **343**:d4519.

27. Garner S: **Disinvestment: the UK experience**. International Society for Pharmacoeconomics and Outcomes Research, 15th Annual European Congress. 2012. http://www.ispor.org/congresses/berlin1112/presentations/W10_Garner.pdf. Accessed 4 Mar 2014.

28. Garner S, Docherty M, Somner J, Sharma T, Choudhury M, Clarke M, Littlejohns P: **Reducing ineffective practice: challenges in identifying low-value health care using Cochrane systematic reviews.** *J Health Serv Res & Policy* 2013, **18**:6-12.

29. Gerdvilaite J, Nachtnebel A: **Disinvestment: Overview of disinvestment experiences and challenges in selected countries**. Ludwig Boltzman Institut fur Health Technology Assessment. 2011. http://eprints.hta.lbg.ac.at/926/#. Accessed 4 Mar 2014.

30. Gershengorn HB, Wunsch H: **Understanding changes in established practice: pulmonary artery catheter use in critically ill patients**. *Crit Care Med* 2013, **41**:2667-2676.

31. Haas JS, Kaplan CP, Gerstenberger EP, Kerlikowske K: **Changes in the use of postmenopausal hormone therapy after the publication of clinical trial results.** *Ann Intern Med* 2004, **140**:184-188.

32. Haas M, Hall J, Viney R, Gallego G: **Breaking up is hard to do: why disinvestment in medical technology is harder than investment**. *Aust Health Rev* 2012, **36**:148-152.

33. Haines T, O'Brien L, McDermott F, Markham D, Mitchell D, Watterson D, Skinner E: **A novel research design can aid disinvestment from existing health technologies with uncertain effectiveness, cost-effectiveness, and/or safety**. *J Clin Epidemiol* 2014, **67**:144-151.

34. Harris E, Mundy L, Hewson K, Jacobsen N: **Disinvestment in Australia and New Zealand**. HealthPACT. 2013. <http://www.health.qld.gov.au/healthpact>. Accessed 4 Mar 2014.

35. Hauptman PJ, Schnitzler MA, Swindle J, Burroughs TE: **Use of nesiritide before and after publications suggesting drug-related risks in patients with acute decompensated heart failure**. *JAMA* 2006, **296**:1877-1884.

36. Henshall C, Schuller T, Mardhani-Bayne L: **Using health technology assessment to support optimal use of technologies in current practice: the challenge of "disinvestment"**. *Int J Technol Assess Health Care* 2012, **28**:203-210.

37. Hersh AL, Stefanick ML, Stafford RS: **National use of postmenopausal hormone therapy: annual trends and response to recent evidence**. *JAMA* 2004, **291**:47-53.

38. Hislop JM: **Societal preferences for health technology disinvestment policy: Views of scottish taxpayers - A qualitative study**. *Value in Health* 2011, **14 (7)**:A356-A357.

39. Hodgetts K, Elshaug AG, Hiller JE: **What counts and how to count it: Physicians constructions of evidence in a disinvestment context**. *Soc Sci Med* 2012, **75**:2191-2199.

40. Hollingworth W, Busby J, Jones H, Sterne J: **Can variation in hospital procedure rates identify candidates for health technology reassessment and disinvestment?** *Value in Health* 2013, **16 (7)**:A470.

41. Howard DH, Kenline C, Lazarus HM, Lemaistre CF, Maziarz RT, McCarthy PL, Jr., Parsons SK, Szwajcer D, Douglas Rizzo J, Majhail NS: **Abandonment of high-dose chemotherapy/hematopoietic cell transplants for breast cancer following negative trial results**. *Health Serv Res* 2011, **46**:1762-1777.

42. Howard DH, Shen YC: **Comparative effectiveness research, technological abandonment, and health care spending**. *Adv Health Econ Health Serv Res* 2012, **23**:103-121.

43. Hsiao FY, Tsai YW, Huang WF: **Changes in physicians' practice of prescribing cyclooxygenase-2 inhibitor after market withdrawal of rofecoxib: a retrospective study of physician-patient pairs in Taiwan**. *Clin Ther* 2009, **31**:2618-2627.

44. Huang WF, Tsai YW, Hsiao FY, Liu WC: **Changes of the prescription of hormone therapy in menopausal women: an observational study in Taiwan**. *BMC Public Health* 2007, **7**:56.

45. Hughes DA, Ferner RE: **New drugs for old: disinvestment and NICE**. *BMJ* 2010, **340**:c572.

46. Ibargoyen-Roteta N, Gutierrez-Ibarluzea I, Asua J, Benguria-Arrate G, Galnares-Cordero L: **Scanning the horizon of obsolete technologies: possible sources for their identification**. *Int J Technol Assess Health Care* 2009, **25**:249-254.

47. Ibargoyen-Roteta N, Gutierrez-Ibarluzea I, Asua J: **Guiding the process of health technology disinvestment**. *Health Policy* 2010, **98**:218-226.

48. Ioannidis JP: **In reply II-reversal of medical practices**. *Mayo Clinic Proc* 2013, **88**:1184.

49. Ioannidis JPA: **Contradicted and initially stronger effects in highly cited clinical research**. *JAMA* 2005, **294**:218-228.

50. Joshi NP, Stahnisch FW, Noseworthy TW: **Reassessment of Health Technologies: Obsolescence and Waste**. Canadian Agency for Drugs & Technologies in Health. 2009. <https://www.cadth.ca/reassessment-health-technologies-obsolescence-and-waste> (accessed October 22, 2013).

51. Karnon J, Carlton J, Czoski-Murray C, Smith K: **Informing disinvestment through cost-effectiveness modelling: Is lack of data a surmountable barrier?** *Applied Health Economics and Health Policy* 2009, **7**:1-9.

52. Kelly M: **Public Health Programmes and Interventions and Disinvestment**. National Institute for Health and Care Excellence. 2006. https://www.nice.org.uk/proxy/?sourceUrl=http%3A%2F%2Fwww.nice.org.uk%2FniceMedia%2Fpdf%2Fsmt%2F040406item5.pdf. Accessed 4 Mar 2014.

53. Kiechle FL, Arcenas RC, Rogers LC: **Establishing benchmarks and metrics for disruptive technologies, inappropriate and obsolete tests in the clinical laboratory**. *Clin Chim Acta* 2014, **427**:131-136.

54. Koo KK, Sun JC, Zhou Q, Guyatt G, Cook DJ, Walter SD, Meade MO: **Pulmonary artery catheters: evolving rates and reasons for use**. *Crit Care Med* 2011, **39**:1613-1618.

55. Kowalczyk KJ, Levy JM, Caplan CF, Lipsitz SR, Yu H-y, Gu X, Hu JC: **Temporal National Trends of Minimally Invasive and Retropubic Radical Prostatectomy Outcomes from 2003 to 2007: Results from the 100% Medicare Sample**. *European Urology* 2012, **61**:803-809.

56. Krol N, Wensing M, Haaijer-Ruskamp F, Muris JWM, Numans ME, Schattenberg G, Balen J, Grol R: **Patient-directed strategy to reduce prescribing for patients with dyspepsia in general practice: a randomized trial**. *Aliment Pharmacol Ther* 2004, **19**:917-922.

57. Kulawik D, Sands JJ, Mayo K, Fenderson M, Hutchinson J, Woodward C, Gore S, Asif A: **Focused vascular access education to reduce the use of chronic tunneled hemodialysis catheters: results of a network quality improvement initiative**. *Semin Dial* 2009, **22**:692-697.

58. Lawton B, Rose S, McLeod D, Dowell A: **Changes in use of hormone replacement therapy after the report from the Women's Health Initiative: cross sectional survey of users**. *BMJ* 2003, **327**:845-846.

59. **Medical Reversal.** Life in the Fast Lane. 2014. <http://lifeinthefastlane.com/education/ccc/medical-reversal> Accessed 3 Jun 2014.

60. Leggett LE, Mackean G, Noseworthy TW, Sutherland L, Clement F: **Current status of health technology reassessment of non-drug technologies: survey and key informant interviews**. *Health Res Policy Syst* 2012, **10**:38.

61. Leggett L, Noseworthy TW, Zarrabi M, Lorenzetti D, Sutherland LR, Clement FM: **Health technology reassessment of non-drug technologies: current practices**. *Int J Technol Assess Health Care* 2012, **28**:220-227.

62. Leng G: **Introduction of new disinvestment programmes**. National Institute for Health & Care Excellence. 2006. https://www.nice.org.uk/proxy/?sourceUrl=http%3A%2F%2Fwww.nice.org.uk%2FniceMedia%2Fpdf%2Fsmt%2F210206item3.pdf. Accessed 4 Mar 2014.

63. Levin L: **Disinvestment strategies based on evidence guided adoption and obsolescence of technologies: the Ontario experience**. Canadian Foundation for Healthcare Improvement 2011. http://www.cfhi-fcass.ca/Libraries/CEO_Forum_files/LevinENG.sflb.ashx. Accessed 4 Mar 2014.

64. Luetmer MT, Kallmes DF: **Have referral patterns for vertebroplasty changed since publication of the placebo-controlled trials?** *Am J Neuroradiol* 2011, **32**:647-648.

65. MacKean G, Noseworthy T, Elshaug AG, Leggett L, Littlejohns P, Berezanski J, Clement F: **Health technology reassessment: the art of the possible**. *Int J Technol Assess Health Care* 2013, **29**:418-423.

66. Majumdar SR, Inui TS, Gurwitz JH, Gillman MW, McLaughlin TJ, Soumerai SB: **Influence of physician specialty on adoption and relinquishment of calcium channel blockers and other treatments for myocardial infarction**. *J Gen Intern Med* 2001, **16**:351-359.

67. Majumdar SR, Almasi EA, Stafford RS: **Promotion and prescribing of hormone therapy after report of harm by the Women's Health Initiative**. *JAMA* 2004, **292**:1983-1988.

68. Massatti RR, Sweeney HA, Panzano PC, Roth D: **The de-adoption of innovative mental health practices (IMHP): why organizations choose not to sustain an IMHP**. *Adm Policy Mental Health* 2008, **35**:50-65.

69. Mortimer D: **Reorienting programme budgeting and marginal analysis (PBMA) towards disinvestment**. *BMC Health Serv Res* 2010, **10**:288.

70. Moscucci M: **Medical reversal, clinical trials, and the "late" open artery hypothesis in acute myocardial infarction**. *Arch of Intern Med* 2011, **171**:1643-1644.

71. Moynihan RN: **A healthy dose of disinvestment.** *Med J Austr* 2012, **196**:158-158.

72. Murphy DJ, Needham DM, Netzer G, Zeger SL, Colantuoni E, Ness P, Pronovost PJ, Berenholtz SM: **RBC Transfusion Practices Among Critically Ill Patients**. *Crit Care Med* 2013, **41**:2344-2353.

73. Naylor CD: **The complex world of prescribing behavior**. *JAMA* 2004, **291**:104-106.

74. Nieuwlaat R, Schwalm JD, Khatib R, Yusuf S: **Why are we failing to implement effective therapies in cardiovascular disease?** *European Heart J* 2013, **34**:1262-1269.

75. Noseworthy T, Clement F: **Health technology reassessment: scope, methodology, & language**. *Int J Technol Assess Health Care* 2012, **28**:201-202.

76. Nuti S, Vainieri M, Bonini A: **Disinvestment for re-allocation: A process to identify priorities in healthcare**. *Health Policy* 2010, **95**:137-143.

77. Paulden M: **Investment and disinvestment of health technologies: The need for two cost-effectiveness thresholds**. *Value in Health* 2012, **15 (4)**:A33.

78. Pearson S, Littlejohns P: **Reallocating resources: how should the National Institute for Health and Clinical Excellence guide disinvestment efforts in the National Health Service?** *J Health Serv Res Policy* 2007, **12**:160-165.

79. Polisena J, Clifford T, Elshaug AG, Mitton C, Russell E, Skidmore B: **Case studies that illustrate disinvestment and resource allocation decision-making processes in health care: a systematic review**. *Int J Technol Assess Health Care* 2013, **29**:174-184.

80. Prasad V, Gall V, Cifu A: **The frequency of medical reversal**. *Arch Intern Med* 2011, **171**:1675-1676.

81. Prasad V, Cifu A: **Medical reversal: why we must raise the bar before adopting new technologies**. *Yale J Biol Med* 2011, **84**:471-478.

82. Prasad V, Cifu A, Ioannidis JPA: **Reversals of established medical practices: evidence to abandon ship**. *JAMA* 2012, **307**:37-38.

83. Prasad V, Vandross A: **Cardiovascular primary prevention: how high should we set the bar?** *Arch of Intern Med* 2012, **172**:656-659.

84. Prasad V, Vandross A, Toomey C, Cheung M, Rho J, Quinn S, Chacko SJ, Borkar D, Gall V, Selvaraj S *et al*: **A decade of reversal: an analysis of 146 contradicted medical practices**. *Mayo Clinic Proc* 2013, **88**:790-798.

85. Prasad V, Cifu A: **In reply I-reversal of medical practices**. *Mayo Clinic Proc* 2013, **88**:1183-1184.

86. Prasad V, Ioannidis JP: **Evidence-based de-implementation for contradicted, unproven, and aspiring healthcare practices**. *Implement Sci* 2014, **9**:1.

87. **Towards better patient care: drugs to avoid**. *Prescrire Int* 2013, **22**:108-111.

88. Ross-Degnan D, Soumerai SB, Fortess EE, Gurwitz JH: **Examining product risk in context. Market withdrawal of zomepirac as a case study**. *JAMA* 1993, **270**:1937-1942.

89. Roumie CL, Grogan EL, Falbe W, Awad J, Speroff T, Dittus RS, Elasy TA: **A three-part intervention to change the use of hormone replacement therapy in response to new evidence**. *Ann Intern Med* 2004, **141**:118-125.

90. Setakis E, Leufkens HG, van Staa TP: **Changes in the characteristics of patients prescribed selective cyclooxygenase 2 inhibitors after the 2004 withdrawal of rofecoxib**. *Arthritis Rheum* 2008, **59**:1105-1111.

91. Sindby JE, Brocki BC, Rasmussen BS, Gorst-Rasmussen A, Andreasen JJ: **Efforts to change transfusion practice behaviour and reduce transfusion rates are effective in coronary artery bypass surgery**. *J Cardiothorac Vasc Anesth* 2011, **1**:S46.

92. Siontis GCM, Tatsioni A, Katritsis DG, Ioannidis JPA: **Persistent reservations against contradicted percutaneous coronary intervention indications: citation content analysis**. *Am Heart J* 2009, **157**:695-701.

93. Smalley W, Shatin D, Wysowski DK, Gurwitz J, Andrade SE, Goodman M, Chan KA, Platt R, Schech SD, Ray WA: **Contraindicated use of cisapride: impact of food and drug administration regulatory action**. *JAMA* 2000, **284**:3036-3039.

94. Stafford RS, Radley DC: **National trends in antiobesity medication use**. *Arch Intern Med* 2003, **163**:1046-1050.

95. Stafford RS, Furberg CD, Finkelstein SN, Cockburn IM, Alehegn T, Ma J: **Impact of clinical trial results on national trends in alpha-blocker prescribing, 1996-2002**. *JAMA* 2004, **291**:54-62.

96. Sukel MP, van der Linden MW, Chen C, Erkens JA, Herings RM: **Large-scale stopping and switching treatment with COX-2 inhibitors after the rofecoxib withdrawal**. *Pharmacoepidemiol Drug Saf* 2008, **17**:9-19.

97. Sun SX, Lee KY, Bertram CT, Goldstein JL: **Withdrawal of COX-2 selective inhibitors rofecoxib and valdecoxib: impact on NSAID and gastroprotective drug prescribing and utilization**. *Curr Med Res Opin* 2007, **23**:1859-1866.

98. Street JM, Hennessy SE, Watt AM, Hiller JE, Elshaug AG: **News and social media: windows into community perspectives on disinvestment**. *Int J Technol Assess Health Care* 2011, **27**:376-383.

99. Tatsioni A, Bonitsis NG, Ioannidis JPA: **Persistence of contradicted claims in the literature**. *JAMA* 2007, **298**:2517-2526.

100. Tatsioni A, Siontis GCM, Ioannidis JPA: **Partisan Perspectives in the Medical Literature: A Study of High Frequency Editorialists Favoring Hormone Replacement Therapy**. *J Gen Intern Med* 2010, **25**:914-919.

101. Thiebaud P, Patel BV, Nichol MB: **Impact of rofecoxib withdrawal on cyclooxygenase-2 utilization among patients with and without cardiovascular risk**. *Value Health* 2006, **9**:361-368.

102. Voorn VM, Marang-van de Mheen PJ, So-Osman C, Vlieland TP, Koopman-van Gemert AW, Nelissen RG, van Bodegom-Vos L, Group LS, Brand A, Engberts DP *et al*: **Designing a strategy to implement cost-effective blood transfusion management in elective hip and knee arthroplasties: a study protocol**. *Implement Sci* 2012, **7**:58.

103. Warner JL, Yang P, Alterovitz G: **Reversal of medical practices**. *Mayo Clinic Proc* 2013, **88**:1182-1183.

104. Watt AM, Hiller JE, Braunack-Mayer AJ, Moss JR, Buchan H, Wale J, Riitano DE, Hodgetts K, Street JM, Elshaug AG *et al*: **The ASTUTE Health study protocol: deliberative stakeholder engagements to inform implementation approaches to healthcare disinvestment**. *Implement Sci* 2012, **7**:101.

105. Watt AM, Willis CD, Hodgetts K, Elshaug AG, Hiller JE: **Engaging clinicians in evidence-based disinvestment: role and perceptions of evidence**. *Int J Technol Assess Health Care* 2012, **28**:211-219.

106. Wiener RS, Welch HG: **Trends in the use of the pulmonary artery catheter in the United States, 1993-2004**. *JAMA* 2007, **298**:423-429.

107. Williams D, Singh M, Hind C: **The effect of the withdrawal of rofecoxib on prescribing patterns of COX-2 inhibitors in Scotland**. *Br J Clin Pharmacol* 2006, **62**:366-368.

108. Williams IP, Bryan S: **Cost-effectiveness analysis and formulary decision making in England: findings from research**. *Soc Sci Med* 2007, **65**:2116-2129.

109. Xie F, Petitti DB, Chen W: **Prescribing patterns for antihypertensive drugs after the Antihypertensive and Lipid-Lowering Treatment to Prevent Heart Attack Trial: report of experience in a health maintenance organization**. *Am J Hypertens* 2005, **18**:464-469.
